# Comprehensive Genomic Profiling of *NF2*-Mutated Kidney Tumors Reveals Potential Targets for Therapy

**DOI:** 10.1093/oncolo/oyad040

**Published:** 2023-03-14

**Authors:** Sean M Hacking, Dean Pavlick, Yihong Wang, Benedito A Carneiro, Matthew Mullally, Shaolei Lu, Mariana Canepa, Gennady Bratslavsky, Joseph Jacob, Andrea Necchi, Philippe E Spiess, Li Wang, Evgeny Yakirevich, Jeffrey Ross

**Affiliations:** Department of Pathology and Laboratory Medicine, Rhode Island Hospital, Warren Alpert Medical School of Brown University, Providence, RI, USA; Foundation Medicine, Inc., Cambridge, MA, USA; Department of Pathology and Laboratory Medicine, Rhode Island Hospital, Warren Alpert Medical School of Brown University, Providence, RI, USA; Lifespan Cancer Institute, Legorreta Cancer Center at Brown University, Providence, RI, USA; Department of Pathology and Laboratory Medicine, Rhode Island Hospital, Warren Alpert Medical School of Brown University, Providence, RI, USA; Department of Pathology and Laboratory Medicine, Rhode Island Hospital, Warren Alpert Medical School of Brown University, Providence, RI, USA; Department of Pathology and Laboratory Medicine, Rhode Island Hospital, Warren Alpert Medical School of Brown University, Providence, RI, USA; Department of Urology, State University of New York (SUNY), Upstate Medical University, Syracuse, NY, USA; Department of Urology, State University of New York (SUNY), Upstate Medical University, Syracuse, NY, USA; Department of GU Medical Oncology, San Raffaele University, Milan, Italy; Department of Genitourinary Oncology, Moffitt Cancer Center, Tampa, FL, USA; Department of Pathology and Laboratory Medicine, Rhode Island Hospital, Warren Alpert Medical School of Brown University, Providence, RI, USA; Department of Pathology and Laboratory Medicine, Rhode Island Hospital, Warren Alpert Medical School of Brown University, Providence, RI, USA; Foundation Medicine, Inc., Cambridge, MA, USA

**Keywords:** *NF2*, genomic alteration, kidney tumors, renal cell carcinoma, comprehensive genomic profiling, PD-L1

## Abstract

Genomic alterations (GA) in *NF2* tumor-suppressor gene have been associated with aggressive behavior in kidney tumors. We used comprehensive genomic profiling (CGP) to evaluate the frequencies of *NF2* GA in histologic subtypes of kidney tumors and co-occurring GA in other genes and biomarkers. Advanced kidney tumors included 1875 clear cell (ccRCC), 405 papillary (pRCC), 108 chromophobe (chRCC), 171 sarcomatoid (sRCC), 61 collecting duct (cdRCC), 49 medullary (mRCC), 134 unclassified (uRCC), 906 urothelial carcinoma of renal pelvis (UC), and 147 Wilms tumors underwent hybrid-capture based CGP to evaluate all classes of GA. 192 (4.9%) of kidney tumors featured *NF2* GA which were predominantly structural variant mutations (89%), followed by copy number alterations (9%). Gender and age were similar between *NF2*-mutant (*NF2*mut) and *NF2*-wild type (*NF2*wt) cohorts with male preponderance. *NF2* GA frequency was highest in cdRCC (30%), sRCC (21%), uRCC (15%), and pRCC (12%) while lowest in ccRCC (3%), UC (3%) Wilms tumor (1%), and chRCC (0%). *NF2* mutational status was associated with loss of Ch 22 (*P* < .001). *NF2*mut RCC harbored co-occurring GA including *CDKN2A*, *CDKN2B*, *SETD2*, and *BAP1*. *VHL*, *PBRM1*, *PTEN*, and *FGFR3* GA were significantly more frequent in *NF2*wt than in *NF2*mut tumors. MTOR pathway GAs were uncommon in *NF2*mut tumors. No *NF2* mutated RCC featured MSI-high or high TMB. sRCC was associated with high PD-L1 expression. PD-L1 SP142 tumoral (*P* = .04) and immune cells (*P* = .013) were more frequent in *NF2*mut as compared to *NF2*wt group. Among histologic subtypes of RCC, cdRCC, sRCC, pRCC, and uRCC are enriched in *NF2* GA. Co-occurrent GA in *CDKN2A/B*, *SETD2*, and *BAP1* may represent potential therapeutic targets. Higher level of PD-L1 expression in *NF2*mut cohort suggests that these tumors might be sensitive to immune checkpoint inhibitor therapies.

## Teaser

In this study, comprehensive genomic profiling (CGP) was used to evaluate the frequency of NF2 genomic alterations in histologic subtypes of kidney tumors and co-occurring genomic alterations in other genes and biomarkers.

**Implications for Practice**

In this study, 192 (4.9%) kidney tumors that featured *NF2* genomic alterations (GA) were found. Among histologic subtypes of renal cell carcinoma (RCC), aggressive variants, such as collecting duct RCC, sarcomatoid RCC, papillary RCC, and unclassified RCC were found to be enriched in *NF2* GA (30%, 21%, 12%, and 15%, respectively). In these RCC subtypes, *NF2* genomic alteration appears to serve as predominant driver mutation, with corresponding suppression of additional driver mutations in the MTOR pathway and other targetable kinases. Co-occurrent GA in *CDKN2A/B*, *SETD2*, and *BAP1* may represent potential therapeutic targets. The higher level of PD-L1 expression seen in *NF2*-mutated kidney tumors suggests they may be sensitive to immune checkpoint inhibitor therapies.

## Introduction

Kidney tumors are heterogeneous and categorized by distinct histopathological features and genomic alterations.^1^ Renal cell carcinoma (RCC) is a the most common kidney malignancy and is classified into clear cell RCC (ccRCC, 75%), and more rare histologic variants collectively grouped as non-clearcell RCC (nccRCC, 25%). nccRCC include papillary RCC (pRCC, 15%), chromophobe RCC (chRCC, 5%), unclassified RCC (uRCC, 5%), and other rare subtypes such as medullary (mRCC, <1%) and collecting duct (cdRCC, <1%).^1^. Sarcomatoid differentiation (sRCC) is a morphologic change that can be seen in all subtypes and typically portends a poor prognosis.^2^

Molecular profiles have long been known to correlate with histologic kidney cancer subtypes.^3^ There is a well-established genotype-phenotype association between *VHL* alterations and ccRCC, *cMET* protooncogene activation in low grade pRCC, fumarate hydratase (*FH*) inactivating mutations and hereditary leiomyomatosis and RCC (HLRCC) syndrome-associated renal cancer, succinate dehydrogenase (*SDH*)-inactivating mutations and *SDH*-deficient RCC, amongst others.^4^ In the most recent 2022 WHO classification of renal tumors, molecularly driven subtypes have been introduced including SMARCB1-deficient medullary RCC, *TFE***B**-rearranged RCC, *ALK*-rearranged RCC, and *ELOC*-mutated RCC.^5^

Recently, a RCC with *NF2* genomic alteration (GA) gained attention not only due to morphologic features^6,7^ but also due to its association with treatment responses in nccRCC.^6^ In a recent phase II clinical trial of advanced nccRCC, 5 of 6 patients with *NF2* mutations achieved an objective response to multi-targeted tyrosine kinase inhibitor cabozantinib plus human programmed death receptor-1 (PD-1) blocker nivolumab.^8^

*NF2* gene on chromosome 22q encodes the tumor suppressor protein moesin-ezrin-radixin-like protein (merlin), also known as schwannomin important for the function of various mitogenic signaling pathways, including receptor tyrosine kinases (RTKs), Rac/p-21 activated kinase (RAK), mammalian target of rapamycin (mTOR), and the Hippo pathway.^9,10^ Neurofibromatosis type 2 syndrome is caused by heterozygous germline *NF2* loss or inactivation and results in the development of vestibular schwannomas, meningiomas, ependymomas, and ocular disturbances.^11^*NF2* GA have been postulated to defect the *NF2* protein at the interface between the plasma membrane and the cytoskeleton, leading to dysfunction in adhesion, which is essential to cellular development and regeneration.^12,13^

Currently, there is expanding interest in defining predictive and prognostic role of PD-L1 expression for immune checkpoint inhibitors therapies.^14-16^ Across 15 tumor types including RCC, Davis et al. demonstrated PD-L1 was predictive for sensitivity to immune checkpoint blockade in 28.9% of cases, not predictive in 53.3%, and not tested in the remaining 17.8% of cases.^16^ A retrospective study of 306 ccRCC patients revealed PD-L1 (B7-H1) expression to be significantly associated with poorer cancer survival rates (41.9%) when compared to those whose tumors did not express PD-L1 (82.9%).^17^ PD-1-specific therapies including nivolumab^18,19^ and pembrolizumab,^20^ along with the PD-L1 antibody avelumab,^21^ have received FDA approval in metastatic RCC.

In this study, we performed comprehensive genomic profiling (CGP) of a large cohort of 3919 clinically advanced kidney tumors. Our findings demonstrate that *NF2* GA are frequent in nccRCCs, and especially enriched in cdRCC. *NF2-*mutant (*NF2*mut) tumors often harbor *CDKN2A/B*, *SETD2*, and *BAP1* GA, which are potentially amenable to targeted therapies. Higher frequencies of PD-L1 expression in *NF2*mut group suggest that these patients may benefit from immune checkpoint inhibitors.

## Materials and Methods

### Patient Selection

Approval for this study was obtained from the Western Institutional Review Board (Protocol No. 20152817). We reviewed the Foundation Medicine, Inc. (Cambridge, MA) database to retrieve all kidney tumors tested between 2015 and 2021. All cases submitted to Foundation Medicine were reviewed by pathologist with genitourinary expertise. All cases were clinically advanced, and the vast majority were stage IV. These cases were analyzed by CGP and PD-L1 immunohistochemistry (IHC) during routine clinical care. Demographic data were extracted from pathology reports.

This study received approval by the Institutional Review Board at Foundation Medicine, Inc. (Cambridge, MA).

A second validation cohort from The Cancer Genome Atlas (TCGA) included 1486 patients across the following publicly available datasets. Cases of ccRCC were retrieved from (1) The Cancer Genome Atlas, Firehose Legacy; (2) Nat Genet 2014; (3) Beijing Genome Institute, Nat Genet 2012; (4) Dana-Farber Cancer Institute, Science 2019; (5) University of Tokyo, Nat Genet 2013, chRCC; (6) TCGA, Firehose Legacy, pRCC; (7) TCGA, Firehose Legacy, renal non-clear cell carcinoma; (8) Genentech, Nat Genet 2014, uRCC; (9) MSK, Nature 2016, Pediatric Rhabdoid Tumor; (10) TARGET, 2018, Rhabdoid Cancer; (11) BCGSC, Cancer Cell 2016, Pediatric Wilms Tumor; and (12) TARGET, 2018. Care was taken during cohort creation to not select overlapping patients and samples were excluded if they were unprofiled for *NF2*. The bookmark query for this study from CBioPortal is listed here: https://www.cbioportal.org/study?id=62070c1f0934121b56de2448.

### Comprehensive Genomic Profiling

CGP was performed using the FDA-approved FoundationOne CDx assay (Foundation Medicine, Cambridge, MA) in a Clinical Laboratory Improvement Amendments (CLIA)-certified and College of American Pathologists (CAP)-accredited laboratory using previously described methods.^22^ Prior to nucleic acid extraction, hematoxylin and eosin-stained slides were reviewed to confirm the presence of tumor. DNA extracted from formalin-fixed paraffin-embedded tissues underwent hybrid-capture based next generation sequencing using the FoundationOne platform which interrogates all coding exons of 324 cancer-related genes and introns from 31 genes commonly rearranged in cancer. Data were analyzed for all types of genomic alterations, including base substitutions, insertions/deletions, copy number alterations, and gene rearrangements. In addition, variant-level loss of heterozygosity (LOH), tumor mutational burden (TMB), and microsatellite instability (MSI) were determined. TMB was evaluated on up to 1.1 Mb of sequenced DNA, and MSI was assessed from DNA sequencing across 95 loci as previously described.^23,24^ TMB ≥ 20 mutations/Mb was considered High (TMB-High), >10 mutations/Mb considered intermediate (TMB-Int), and 0-9 mutations/Mb to be TMB low (TMB-low).

### Immunohistochemistry

PD-L1 testing was performed according to individual standard of care and clinical requirements. IHC for PD-L1 was performed according to the manufactures instructions and guidelines using the DAKO PD-L1 22C3 PharmDx assay (Agilent Technologies, Santa Clara, CA) or Ventana PD-L1 SP142 companion diagnostics (CDx) assay (Roche, Tucson, AZ) in a CLIA-certified and CAP-accredited reference laboratory (Foundation Medicine, Morrisville, NC).

For DAKO 22C3 PD-L1 assay was evaluated using the tumor proportion score (TPS) of any intensity, and the combined positive score (CPS). PD-L1 expressing tumor cells were categorized as negative (<1%), low positive (1%-49%), or high positive (≥50%). CPS was calculated as the number of PD-L1 stained cells including tumor cells and immune cells, divided by the total number of tumor cells multiplied by 100. For Ventana SP142, evaluation was based on either the proportion of tumor area occupied by PD-L1 expressing tumor-infiltrating immune cells (IC) of any intensity or the percentage of PD-L1 expressing tumor cells (TPS) of any intensity. PDL-1 IC were scored as negative (IC < 1%), low positive (IC ≥ 1%), and high positive (IC ≥ 10%).

Merlin immunohistochemistry was performed using Ventana Discovery XT autostainer (Roche Diagnostics, Indianapolis, IN). Tissue sections were deparaffinized and pretreated in CC1 solution (EDTA, pH8). The primary anti-Merlin antibody (clone D3S3W, rabbit monoclonal, Cell Signaling Technology, Danvers, MA) was used at 1:100 dilution.

### Statistical Analysis

To examine the landscape of genomic biomarkers in our patient cohort, we extracted the top 50 genes with GA and compared these between the *NF2*mut and *NF2*-wild type (*NF2*wt) tumor subsets. Descriptive statistics such as frequencies and percentages were calculated for the GA in each cohort. Statistical analysis was performed using ANOVA, *χ*2 contingency test, or Fisher’s exact test as appropriate. Analysis was performed using SPSS 1.0.0.1508. A critical *P* value of <.05 was used to indicate statistical significance. We also performed Bonferroni correction for multiple testing by dividing the critical *P* value by the number of cooccurring gene comparisons (30), allowing for a modified *P* value of .00167.

## Results

### Clinicopathologic and Molecular Characteristics

The study cohort of 3919 patients included 1875 ccRCC, 405 pRCC, 108 chRCC, 171 sRCC, 61cdRCC, 49 mRCC, 134 uRCC, 906 urothelial carcinoma (UC), and 147 Wilms tumors (Table 1). The median age of the cohort was 62 years (Supplemental Table S1). No significant difference in age of the patients was observed between ccRCC and nccRCC histologic types except patients with mRCC and Wilms tumor. The patients with mRCC and Wilms tumor were younger (median 27 and 6 years, respectively) as compared to other RCC subtypes. In all histologic subtypes except Wilms tumor male patients outnumbered the female ones. The age of patients with *NF2*mut tumors was 60 years (15->89; Table 1). There was a male predominance in the *NF2*mut ccRCC, pRCC, cdRCC, and sRCC, while in mRCC and WT the gender was equal, and in UC female patients slightly predominated.

**Table 1.** Clinical, molecular, and immune biomarkers in *NF2-*mutated kidney tumors.

Tumor	ccRCC	pRCC	sRCC	cdRCC	mRCC	uRCC	UC	Wilms
Number of cases with *NF2* GA	55/1820 (3%)	43/362 (12%)	30/141 (21%)	14/47 (30%)	2/47 (4%)	25/172 (15%)	30/885 (3%)	2/145 (1%)
Gender	F35%/M65%	F26%/M74%	F23%/M77%	F36%/M64%	F50%/M50%	F12%/M88%	57%F/43%M	F50%/M50%
Median age years (range)	63 (31-83)	61 (23-78)	58 (26-78)	58 (32-72)	40 (15-65)	58 (35->89)	67 (36->89)	26 (20-30)
GA/tumor	4.5	3.1	5.1	2.7	2.0	3.6	11.4	3
*NF2* zygosity status								
SV homozygous	29	33	15	10	0	19	10	1
SV heterozygous	7	1	1	0	1	1	4	1
SV unknown	10	6	8	2	1	4	7	0
CN	8	2	6	1	0	0	0	0
RE	1	1	0	1	0	1	0	0
*NF2* GA Type								
Substitutions	18	22	13	4	1	12	12	2
Ins/del	28	18	11	8	1	12	9	0
Loss	8	2	6	1	0	0	0	0
RE	1	1	0	1	0	1	0	0
Most common co-alterations	*VHL* substitution (44%) *CDKN2A* loss (35%) *CDKN2B* loss (29%) *SETD2* indel (27%) *VHL* indel (24%) *MTAP* loss (11%)	*CDKN2A* loss (19%) *FH* substitution (14%) *CDKN2B* loss (12%) *SETD2* (12%) *BAP1* substitution (9%) *KMT2D* indel (7%)	*CDKN2A* loss (57%) *CDKN2B* loss (53%) *VHL* indel (30%) *TP53* (27%) *BAP1* indel (13%) *MTAP* loss (13%)	*CDKN2A* loss (14%) *SETD2* substitution (14%) *CDKN2B* loss (7%) *BAP1* indel (7%) *MTAP* loss (7%) *PBMR1* indel (7%)	*FBXW7* substitution (100%) *FAT1* indel (50%)	*CDKN2A* loss (32%) *CDKN2B* loss (24%) *SETD2* indel (20%) *TERT* substitution (12%) *MTAP* loss (12%) *PBRM1* indel (12%)	*TERT* substitution (37%) *TP53* substitution (33%) *CDKN2A* loss (20%) *CDKN2B* loss (20%) *HRAS* substitution (17%) *MTAP* loss (13%)	*KMT2C* substitution (50%) *FBXW7* substitution (50%) *ASXL1* indel (50%) *FUBP1* indel (50%)
MSI-high total	0	0	0	0	0	0	2	0
TMB low	47	37	25	13	2	21	13	1
TMB int	8	6	5	1	0	4	2	1
TMB high	0	0	0	0	0	0	6	0

Abbreviations: ccRCC, clear cell RCC; cdRCC, collecting duct; chRCC, chromophobe RCC; CNA, copy number alteration; GA, genomic alteration; MSI, microsatellite instable; mRCC, medullary; pRCC, papillary RCC; RE, rearrangement; sRCC, sarcomatoid; SV, structural variation; TMB, tumor mutational burden; UC, urothelial carcinoma; uRCC, unclassified; Wilms, Wilms tumor.

One hundred ninety-two of kidney tumors featured *NF2* GA (4.9%), while 3727 (95.1%) did not. *NF2* GA frequency was highest in cdRCC (30%) and sRCC (21%) and lowest in ccRCC (3%) and UC (3%) (Table1). Of note, in cdRCC most common GA were involving *NF2* gene. No *NF2* GA were identified in chRCC and therefore this cohort was excluded from further analysis. The most common type of GA in the *NF2* gene was structural variation mutations (89%), followed by copy number alterations (homozygous deletions and amplifications) (9%) and gene rearrangements (2%). Loss of chromosome 22q harboring the *NF2* gene was found in the majority (79%) of *NF2*mut tumors. Overall, 69% of *NF2*mut specimens were under LOH, either with one mutant allele remaining or with multiple copies of mutant allele (homozygous mutations), 9% were heterozygous mutations, and zygosity was unknown in 22%. All *NF2* GA were predicted to be inactivating based on disruption of the FERM domain (amino acids 22-311), which includes in-frame deletions that disrupt the Paxillin-binding region (aa 50-70) of the FERM domain2 as well as the C-terminal region (amino acids 506-547).

**Figure 1.**
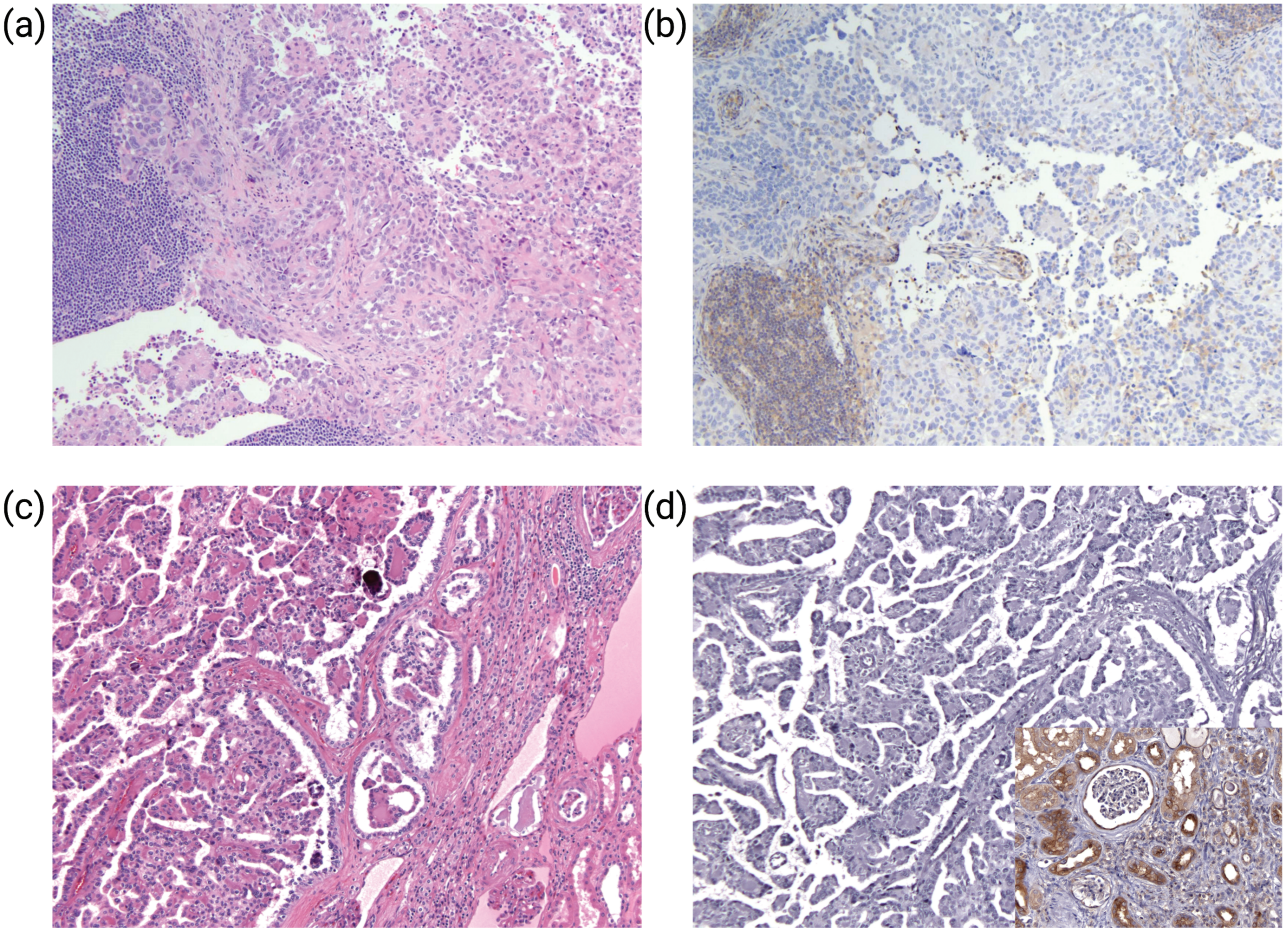
Immunohistochemical analysis of merlin expression in *NF2*-mutated RCC. Representative histologic sections of tumors (H&E) show solid and papillary architecture with small cells clustering around hyaline material and forming micropapillae surrounded by larger cells and scattered calcifications (**a, c**). Negative staining of tumor cells for merlin (**b, d**). The inflammatory cell (**b**, lower left) renal tubules and Bowman capsule (**d**, inset) show retained merlin immunoreactivity.

To confirm that *NF2* GA result in protein loss, we evaluated merlin expression by immunohistochemistry in 2 cases in which tissue was available. Both tumors demonstrated complete loss of merlin expression (Fig. 1). In contrast, inflammatory cells and non-neoplastic kidney tissue adjacent to tumors showed retained merlin expression in renal tubules and Bowman capsule (Fig. 1d, inset).

Analysis of co-mutated genes revealed that *VHL* was the most common co-altered gene in *NF2*mut ccRCC. Deletion of cyclin-dependent kinase inhibitor *CDKN2A* was the most common co-alteration in pRCC (19%), sRCC (57%), cdRCC (14%), and uRCC (32%). *CDKN2B* GA co-occurred with *CDKN2A* in slightly lower frequencies. *FBXW7* was the most altered gene in mRCC (100%). *TERT* was the most commonly co-altered GA in UC (37%). The most commonly co-mutated genes across kidney tumor subtypes can be found in Supplemental Fig. S1.

**Figure 2.**
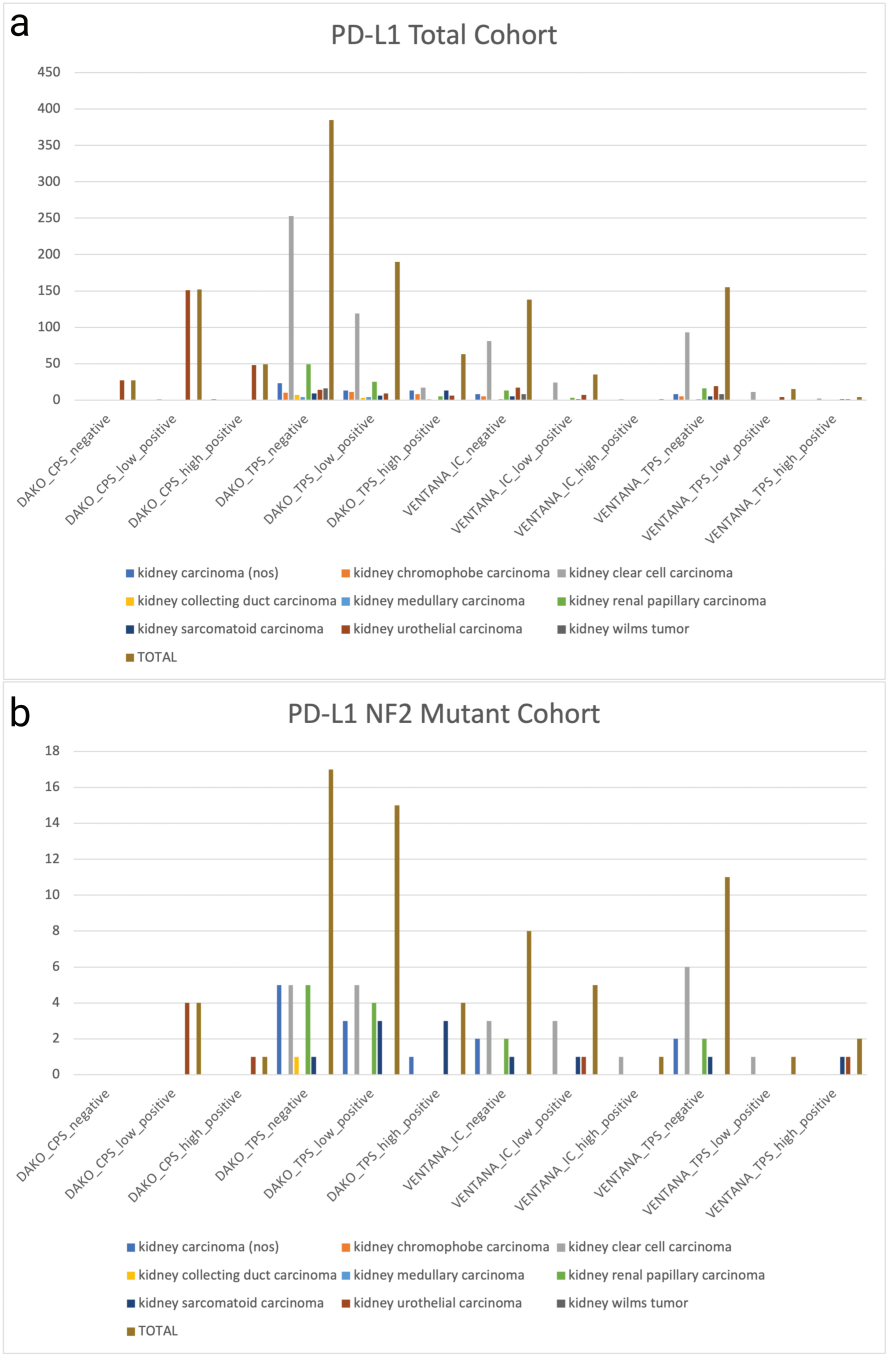
PDL1 scoring with DAKO PD-L1 22C3 and Ventana PD-L1 SP142 in the total cohort (**a**) and *NF2*-mutated cohort (**b**). TPS, tumor proportion score; CPS, combined positive score; IC, immune cells.

Two *NF2*mut kidney tumors featured MSI high and 6 featured TMB high status, although exclusively in UC. Variations in PD-L1 positivity were found across different kidney tumor types (Fig. 2). sRCC was found to be associated with strong positivity for PD-L1 expression in tumor cells (TPS) with both DAKO SP22C3 (43%) and Ventana SP142 (50%). One uRCC case was found to have strong SP22C3 TPS staining (11%), while one case of ccRCC (14%) and one case of UC (20%) were found to have a high IC (SP142), and one case of UC (20%) was found to have a high CPS (SP22C3).

The total number of GA per tumor in the *NF2*mut cohort including *NF2* GA and other co-occurring GA was 4.74, for a total of 911 GA. Seven hundred twenty-five GA were co-occurring including 331 base substitutions (46%), 182 insertion/deletions (25%), 141 homozygous deletions (19%), 41 amplifications (6%), and 30 gene rearrangements (4%).

**Figure 3.**
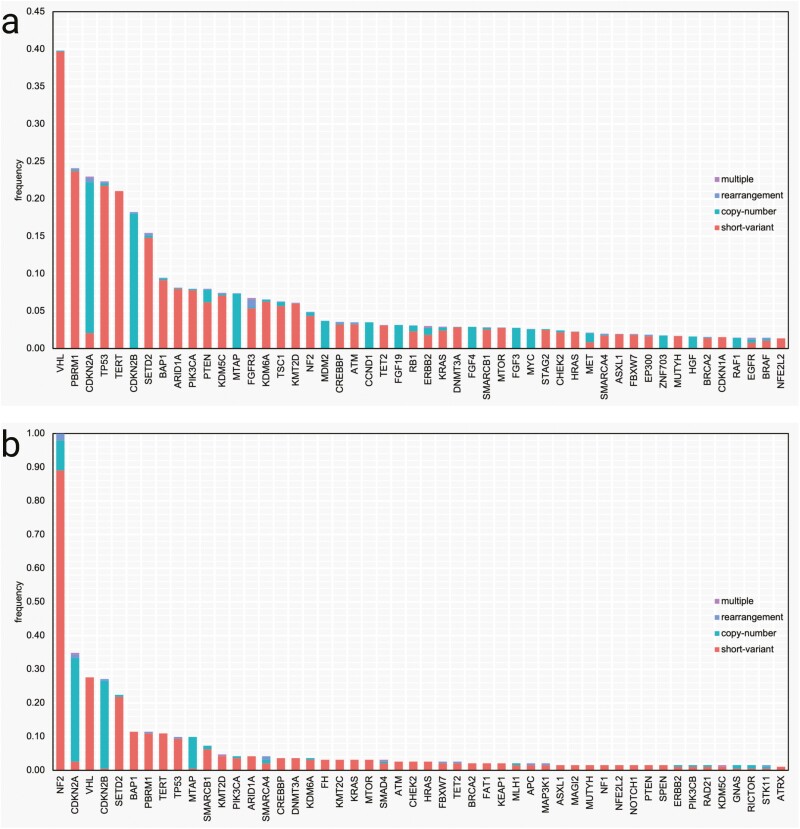
Frequency of pathogenic gene mutations in the total cohort (**a**) and *NF2*-mutated kidney tumors (**b**).

Genomic landscape showing co-mutation plots of the top 50 genes with GA in total cohort and *NF2*mut subset can be seen in Fig. 3. The 4 most common GA in the total disease cohort were: *VHL*, *PBRM1*, *CDKN2A*, and *TP53*; which contrasted with the *NF2*mut disease cohort: *NF2*, *CDKN2A*, *VHL*, and *CDKN28*.

**Figure 4.**
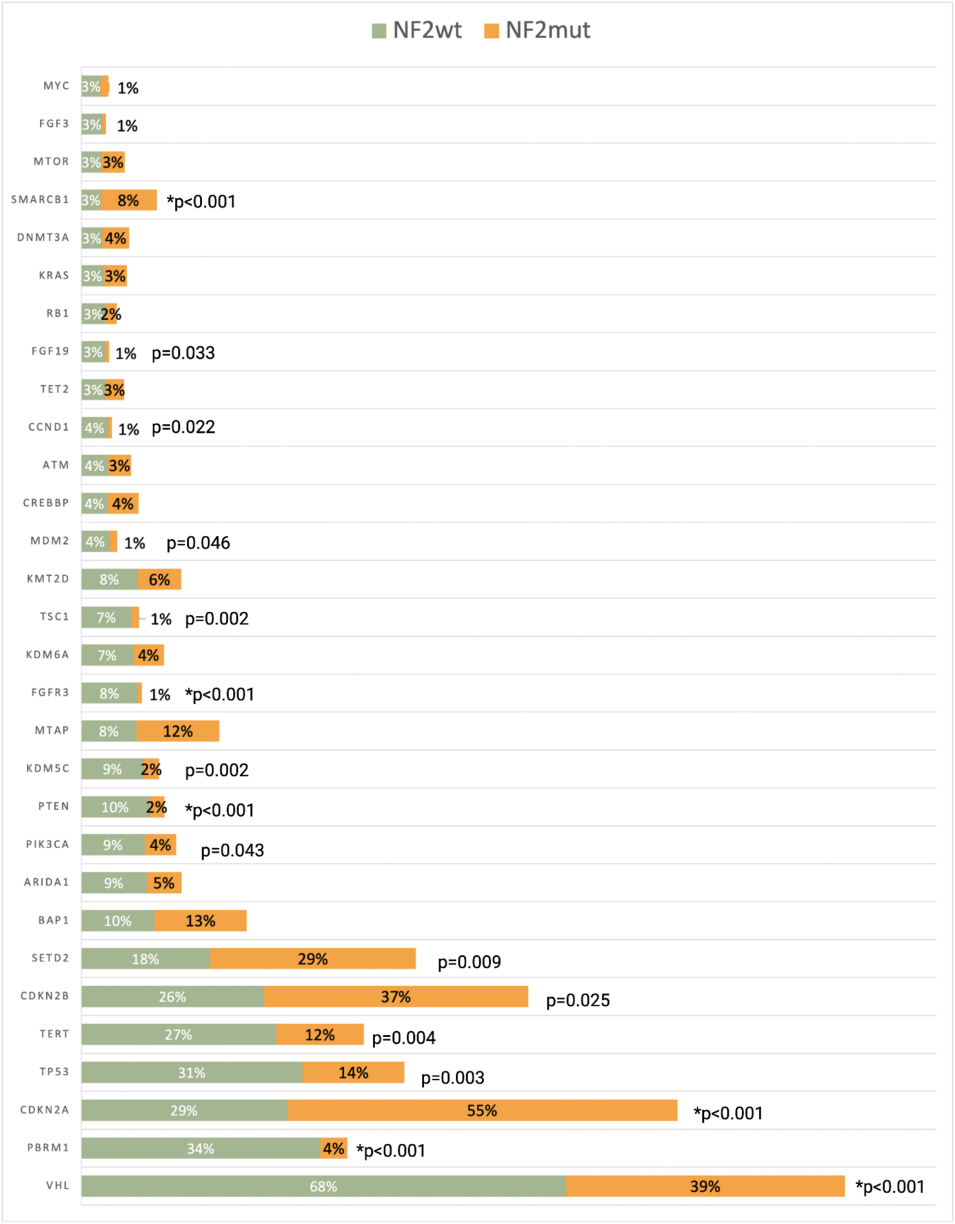
Frequency of top 30 co-occurring genomic alterations (GA) between the *NF2*wt and *NF2*mut tumors. *Significant based on a modified *P* value of .00167 following Bonferroni correction.

Of the top 50 genes in the total cohort, 30 were genes with shared GA in the *NF2*wt and *NF2*mut cohorts. The frequencies of 16 of the 30 shared genes were significantly different between the *NF2*wt and *NF2*mut subsets (Fig. 4). The following GA were found to be enriched in *NF2*mut tumors: *CDKN2A* (*P* < .001), *CDKN2B* (*P* = .025), *SETD2* (*P* = .009), and *SMARCB1* (*P* < .001). In contrast, *NF2*wt tumors featured GA in *VHL* (*P* < .001), *PBRM1* (*P* < .001), *TP53* (*P* = .003), *TERT* (*P* = .004), *PIK3CA* (*P* = .043), *PTEN* (*P* < .001), *KDM5C* (*P* = .002), *FGFR3* (*P* < .001), *TSC1* (*P* = .002), *MDM2* (*P* = .046), *CCND1* (*P* = .022), and *FGF19* (*P* = .033). The remaining GA were not found to be associated with *NF2* mutational status. Only rare co-occurring mutations were identified in mTOR pathway in *NF2*mut tumors, including *PIK3CA* (4%), *MTOR* (3%), *TSC1* (1%), and *PTEN* (2%). Following Bonferroni correction and a modified *P* value of .00167, only *CDKN2A* (*P* < .001), *SMARCB1* (*P* < .001), *VHL* (*P* < .001), *PBRM1* (*P* < .001), *PTEN* (*P* < .001), and *FGFR3* (*P* < .001) were found to be significant.

The results for comparisons between *NF2*wt and *NF2*mut cohorts are presented in Table 2. TMB was not associated with *NF2* mutational status (*P* = .619). Chromosome 22 was more likely to be lost in the *NF2*mut cohort when compared to *NF2*wt tumors (*P* < .001). There was no association between MSI and *NF2* mutational status (*P* = .651).

**Table 2.** Comparisons between *NF2*wt and *NF2*mut cohorts.

Patient characteristics	*NF2* wild type (*n* = 3727)	*NF2* mutant (*n* = 192)	*P* value
Age, years			—
Median	62	60	
Gender			
Male	2504	133	.548^b^
Female	1223	59	
TMB (mutations/Mb)			.619^b^
TMB-high	89	6	
TMB-int	603	27	
TMB-low	3035	159	
Ch 22 status			**<.001** ^b^
Lost	528	89	
Retained	1770	33	
MSI			.651^b^
MSI-H	27	2	
MSS	3276	168	
PD-L1 DAKO 22C3			
TPS positive	59	4	.228^a^
TPS low positive	175	15	
TPS negative	368	17	
CPS positive	48	1	1.000^b^
CPS low positive	148	4	
CPS negative	27	0	
PD-L1 Ventana SP142			
TPS positive	2	2	**.040** ^b^
TPS low positive	14	1	
TPS negative	144	11	
IC positive	0	1	**.013** ^b^
IC low positive	30	5	
IC negative	130	8	

^a^ *χ*2 contingency test.

^b^Fisher’s exact test.

Bolded values are significant based on a *P* value of <.05.

Abbreviations: CPS, combined positive score; IC, immune cells; MSI, microsatellite instable; MSS, microsatellite stable; TMB, tumor mutational burden; TPS, tumor proportion score.

Analysis of PD-L1 expression revealed no difference in PD-L1 22C3 assay TPS (*P* = .228) and CPS (*P* = 1.00) between *NF2*wt and *NF2mut* groups. However, PD-L1 SP142 assay TPS (*P* = .040) and IC (*P* = .013) were found more frequent in *NF2*mut kidney tumors.

### Findings from the Combined TCGA Cohort

**Figure 5.**
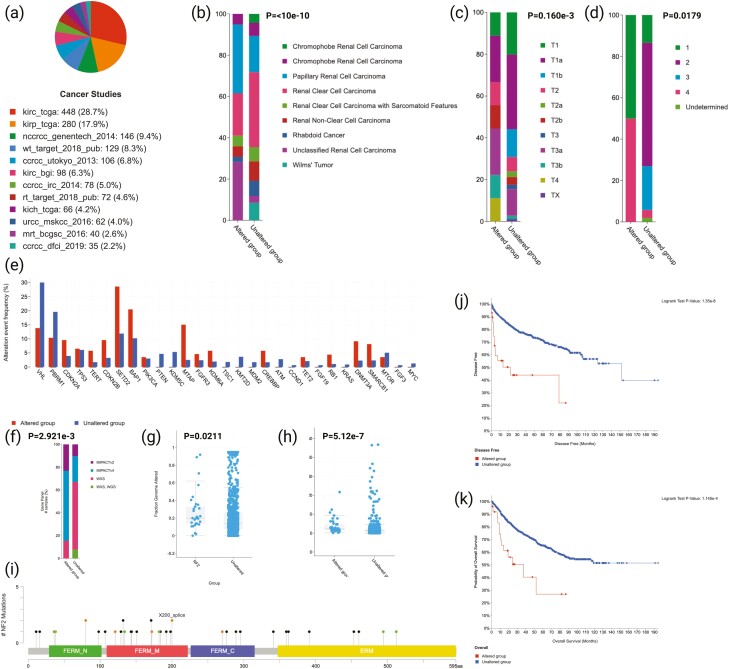
Findings from the TCGA cohort. (**a**) Breakdown of patients according to corresponding originating cancer study. (**b**) Histologic subtypes according to *NF2* mutational status. (**c**) AJCC pathologic stage according to *NF2* mutational status. (**d**) Histologic grade according to *NF2* mutational status. (**e**) The top 30 co-altered genes found in the Foundation Medicine cohort were analyzed and demonstrated in relation to *NF2* mutational status. (**f**) *NF2* mutational status in relation to gene panels. (**g**) The fraction of genomic alteration according to *NF2* mutational status (**h**) TMB according to *NF2* mutational status. (**i**) Mutation diagram circles are colored with respect to the corresponding mutation types. In case of different mutation types at a single position, color of the circle is determined with respect to the most frequent mutation type. Mutation types and corresponding color codes are as follows: green, missense mutations; black, truncating mutations; red, inframe mutations; orange, splice mutations; purple, fusion mutations; pink, other mutations (for colour figure refer to online version). (**j**) Kaplan-Meier for disease-free-survival between *NF2*mut and WT groups. (**k**) Kaplan-Meier for overall survival between *NF2*mut and *NF2*wt groups.

In the TCGA validation cohort, 35/1486 (2.4%) kidney tumors harbored *NF2* GA and 37 types of different mutations were seen. Briefly, 32 were driver mutations: 24 truncating and 8 splice, while 5 were variant of undetermined significance (VUS), all of which were missense. The breakdown of patients according to corresponding study percentages can be seen in Fig. 5a. Regarding tumor subtype, *NF2* GA were found to be more common in uRCC and pRCC (*P* < 10e-10) as compared to other histologic types (Fig. 5b). High pathologic stage (*P* = .160e-3) and high WHO/ISUP histologic grade (*P* = .179) were characteristic of tumors with *NF2* GA (Fig. 4c, 4d). The top 30 genes found in the Foundation Medicine cohort were analyzed in relation to *NF2* GA (Fig. 4e). The fraction of GA was higher in the *NF2*mut cohort (median = 0.2) compared to *NF2*wt tumors (median = 0.14; *P* = .0211); a finding also seen with TMB (*P* = 5.12e-7; Fig. 4g, 4h). Mutation diagram circles are colored with respect to the corresponding mutation types can be seen in Fig. 4i. Regarding survival, *NF2*mut kidney tumors featured lower disease-free (*P* = 1.35e-8) and overall survival (*P* = 1.145e-4) when compared to the *NF2*wt group (Fig. 4j, 4k). *VHL* was validated as being more commonly mutated in the *NF2*wt cohort (*P* = .0393), while *NF2*mut tumors harbored *SETD2* (*P* < .001) and *BAP1* (*P* = .0344) GA, similarly to Foundation Medicine cohort. Following Bonferroni correction and a modified *P* value of .00167, only *SETD2* was found to be significant. Findings from the validation of the top 30 co-occurring genes can be found in Supplemental Table S2.

## Discussion

In this study, we characterized the genomic landscape of *NF2*-mutated kidney tumors in a large cohort of 3919 cases. Germline *NF2* loss or inactivation is associated with neurofibromatosis type 2 syndrome, which results in the development of bilateral vestibular schwannomas, meningiomas, and ependymomas.^10^ Loss of merlin encoded by *NF2* gene is found also in 40%-60% of sporadic meningiomas.^25^ In addition to tumors of the nervous system, *NF2* GA alterations and merlin inactivation also occur in a large proportion of malignant mesothelioma (MM) patients. *NF2* GA were found as the most frequent GA in asbestos nonexposed patients with a third of the patients carrying *NF2* mutations.^26^*NF2* GA are less frequent in ovarian serous carcinoma, glioblastoma multiforme, breast, colorectal, skin, hepatic, medullary thyroid, prostate cancer, and melanoma.^10^

Inactivating *NF2* GA have been described in spectrum of kidney tumors including aggressive variants such as cdRCC (29%),^27^ pRCC (12%),^28,29^ sRCC (19.2%),^30^ and uRCC (18%),^31^ as well as in more indolent mucinous and spindle cell carcinoma of the kidney.^32^ The frequencies of GA in histologic subtypes of renal tumors in our cohort are similar to previous studies.

Differences in *NF2* GA frequencies between the Foundation Medicine (4.9%) and TCGA (2.4%) cohorts could be secondary to selection bias since most tumors being tested in the Foundation Medicine were advanced stage IV kidney tumors in contrast to the limited number of patients with confirmed stage IV disease in TCGA cohort. Based on a modified *P* value of .00167, only *SETD2* was found to be significant. Many genes trended towards being significant (*CDKN2A*, *CDKN2B*, and *SMARCB1*) but the cohort was small (35 patients).

The relatively high prevalence of *NF2* GA in a subset of nccRCC, lack of other driver genes, and low *NF2* GA frequency (3%) in ccRCC suggest its driving role in the tumorigenesis. Our finding of low incidence of *NF2* mutations in ccRCC is congruent with previous cohort of 220 metastatic ccRCC in RECORD3 study (4%).^33^ Co-occurrence of *NF2* GA with *VHL* mutations in ccRCC cohort suggests that *NF2* GA may be a secondary event in ccRCC, similarly to co-occurrence of *TSC1* and *TSC2* GA in *VHL* driven ccRCC.^34^ In the study of sarcomatoid ccRCC Malouf et al. presented one tumor with deleterious *NF2* mutation in its sarcomatoid component only, suggesting that *NF2* GA may represent a late event in ccRCC with sarcomatoid differentiation.^35^

The significant proportion of *NF2*mut renal tumors in our series have co-occurring inactivating GA in other tumor suppressor genes. These include cell cycle regulator genes *CDKN2A/2B*, chromatin remodeler genes *BAP1*, *SETD2*, and SWI/SNF-related chromatin remodeler *SMARCB1*. In the study of uRCC by Chen et al., *NF2* GA also co-occurred with *SETD2* and *BAP1*, and the occurrence of *SETD2* mutations was significantly higher in uRCC tumors with *NF2* loss than in remaining uRCC tumors (44% vs 9%.).^31^ In recent study of 14 *NF2*-mutated RCC cases, co-occurrence of *NF2* and chromatin modulator *PBRM1* GA was found in 5 (42%) cases.^6^ However, no *NF2*wt group was included in this study for comparison, and the number of cases was relatively small in contrast to our series.^6^ Although *PBRM1* GA were found in 3.6% of *NF2*mut tumors in our study, the prevalence of this alteration was significantly lower as opposed to *NF2*wt group (25.1%).

*NF2* tumor suppressor gene inactivation along with mutations in *CDKN2A/B*, and chromatin modulators *BAP1*, *SETD2*, and *SMARCB1* has been described as driving GA in high-grade/progressive meningioma, and MM similarly to *NF2*mut RCC.^36-38^ All these are highly aggressive tumors refractory to conventional therapies. Our analysis of TCGA data supports aggressive behavior of *NF2*mut renal tumors. Mutations in *CDKN2A/B* were found to be the most associated co-alteration in aggressive *NF2*mut meningiomas, seen in 24% of cases.^37^*SMARCB1* mutations were also found in *NF2*mut intraventricular meningioma.^39^ Recently, a mouse model of MM was generated based upon disruption of the *NF2*, *BAP1*, and *CDKN2A/B* tumor suppressor loci in various combinations as also frequently observed in human MM.^40^ Inactivation of all 3 loci in the mesothelial lining of the thoracic cavity led to a highly aggressive MM that recapitulates the histologic features and gene expression profile observed in human MM.

As all major GA in *NF2*mut RCC are tumor suppressor genes, targeted therapies that exploit abnormal tumor suppressor genes have proven far more difficult as opposed to inhibition of oncoproteins. It is important to mention that the loss or inactivation of *NF2* may have the ability to predict sensitivity to focal adhesion kinase inhibitors, this is based on strong preclinical data from malignant pleural mesothelioma.^41^ Preclinical mouse models of *NF2*mut meningiomas have shown overexpression of the mTOR signaling complex 1 pathway, which can be suppressed by mTOR inhibitors.^42^ Limited preclinical and clinical evidence in vestibular schwannoma suggest possible sensitivity of *NF2*-deficient tumors to the pan-ERBB inhibitor lapatinib.^43^ Similarly, based on limited clinical and preclinical evidence, *NF2* inactivation may predict sensitivity to MEK inhibitors, such as approved agents trametinib and cobimetinib.^44^ Data from a Chinese breast cancer cohort suggest that *NF2* loss-of-function mutations may increase sensitivity to Hippo-targeting strategies.^45^ Targeting the Hippo pathway including downstream effectors YAP/TAZ can be a valid approach in renal tumors as well.^35^ In a preclinical model of *NF2*mut pRCC, inhibition of the YAP1 partner YES1 by dasatinib or sarcatinib led to repression of Hippo transcriptional targets and provided potent antitumor activity.^46^*CDKN2A/B*, *BAP1*, and *SETD2* may also represent potential therapeutic targets, as demonstrated in preclinical studies of other tumor types.^25^ These potential therapeutic strategies warrant further investigation in clinical trials.

Immunotherapy is another potential target for investigation in *NF2*mut RCC, as we demonstrated higher level of PD-L1 expression in *NF2*mut cohort. Expression of PD-L1 on tumor and immune cells appears to impact efficacy of PD-L1 inhibitor pembrolizumab. In the KEYNOTE-427 trial from advanced non-clear cell RCC the response rate was 35.3% with a CPS ≥1 as opposed to 12.1% in patients with CPS less than 1.^47^ Combinations of immune checkpoint inhibitors with TKIs such as cabozantinib and axitinib have higher anti-tumor activity and are currently approved for treatment of metastatic clear cell RCC.^48,49^ Selecting tumors with higher PD-L1 expression such as those with *NF2* GA might expand the benefit of these combinations to non-clear cell RCC. In a phase II trial of cabozantinib that targets MET, AXL, and VEGFR2 plus nivolumab, a human PD-1 blocking antibody, *NF2* GA were found in 19% of unclassified/papillary, and translocation-associated RCC (6). Of note, objective tumor responses were seen in 5/6 patients with tumors harboring *NF2* mutations (6). Although conclusions are limited by small sample size, they suggest that *NF2* GA may predict treatment responses in non-clear cell RCC. Paintal et al. reported 2 cases of *NF2*mut RCC with dramatic response to immune checkpoint inhibitors (ipilimumab/nivolumab).^6^ Our findings of more frequent PD-L1 tumor and immune cell expression in *NF2*mut tumors support that these patients may benefit form immune checkpoint inhibitors.

There are several limitations of this study. First, the study suffers from selection bias, as it includes only samples sent to molecular analysis, and therefore, the results may not be representative of general population. Similar studies, including consecutive unselected cases of kidney tumors, are needed to further characterize RCCs harboring *NF2* GA. Second, although the FoundationOne panel of the 324 genes is quite comprehensive, it is possible that there may be other important genes that were simply not included in the testing panel, thus limiting our findings. Third, epigenetic mechanisms of *NF2* inactivation were not addressed in this study. Comprehensive studies of promoter methylation and epigenetic inactivation of *NF2* gene are needed. Fourth, histology of the *NF2*mut tumors was not evaluated. Argani et al. described a series of histologically distinct *NF2*mut that they termed biphasic hyalinizing psammomatous RCC.^7^ Paintal et al. described common morphologic features of *NF2*mut RCC in a series of 14 cases.^6^ While the individual morphologic features seen in these cases are non-specific in isolation, the presence of the typical morphologic constellation (eosinophilic cytology, high nuclear grade, tubulopapillary architecture, sclerotic stroma, microscopic coagulative necrosis, and psammomatous calcifications) can allow for their prospective identification and triage for confirmatory molecular studies. The utility of ancillary techniques such as immunohistochemical detection of NF2 protein expression is limited.^7,29^*NF2* gene deletion can be detected by fluorescent in situ hybridization in MM^50^; however, this has not been validated in renal tumors. Currently, comprehensive genomic profiling proved to be a reliable platform for detection of *NF2* GA in RCC.

In conclusion, the present study is the largest to characterize genomic findings in *NF2*-mutated kidney tumors. Although these aggressive tumors are driven by tumor-suppressor genes, they harbor potentially targetable genomic alterations. Higher frequencies of PD-L1 expression in *NF2*mut tumors suggest that these patients may benefit from immune checkpoint inhibitors. Further studies and clinical trials implementing these therapies are warranted to confirm the clinical relevance and benefit.

## Supplementary Material

oyad040_suppl_Supplementary_Tables

oyad040_suppl_Supplementary_Figure

## Data Availability

All data generated and analyzed in this study can be provided by the corresponding author upon reasonable request.

## References

[1] Moch H HP , Ulbright TM, Reuter VE. WHO Classification of Tumours of the Urinary System and Male Genital Organs. 4th ed. Vol 8; 2016. https://books.google.com.mx/books/about/WHO_Classification_of_Tumours_of_the_Uri.html?id=qxQyjgEACAAJ&redir_esc=y 10.1016/j.eururo.2016.02.028 26996659

[2] Blum KA , Gupta S, Tickoo SK, et al. Sarcomatoid renal cell carcinoma: biology, natural history and management. Nat Rev Urol. 2020;17(12):659-678. 10.1038/s41585-020-00382-9 33051619 PMC7551522

[3] D’Avella C , Abbosh P, Pal SK, Geynisman DM. Mutations in renal cell carcinoma. Urol Oncol. 2020;38(10):763-773. 10.1016/j.urolonc.2018.10.027 30478013

[4] Pavlovich CP , Schmidt Laura S., Schmidt LS. Searching for the hereditary causes of renal-cell carcinoma. Nat Rev Cancer. 2004;4(5):381-393. 10.1038/nrc1364 15122209

[5] Lobo J , Ohashi R, Amin MB, et al. WHO 2022 landscape of papillary and chromophobe renal cell carcinoma. Histopathology. 2022;81(4):426-438. 35596618 10.1111/his.14700

[6] Paintal A , Tjota MY, Wang P, et al. NF2-mutated renal carcinomas have common morphologic features which overlap with biphasic hyalinizing psammomatous renal cell carcinoma: a comprehensive study of 14 cases. Am J Surg Pathol. 2022;46(5):617-627. 35034039 10.1097/PAS.0000000000001846

[7] Argani P , Reuter VE, Eble JN, et al. Biphasic hyalinizing psammomatous renal cell carcinoma (BHP RCC): a distinctive neoplasm associated with somatic NF2 mutations. Am J Surg Pathol. 2020;44(7):901-916. 10.1097/PAS.0000000000001467 32217839 PMC7350624

[8] Lee CH , Voss MH, Carlo MI, et al. Phase II trial of cabozantinib plus nivolumab in patients with non-clear-cell renal cell carcinoma and genomic correlates. J Clin Oncol. 2022;40(21):2333-2341. 10.1200/JCO.21.01944 35298296 PMC9287282

[9] Beltrami S , Kim R, Gordon J. Neurofibromatosis type 2 protein, NF2: an uncoventional cell cycle regulator. Anticancer Res. 2013;33(1):1-11. 23267122 PMC3725758

[10] Petrilli AM , Fernández-Valle C. Role of Merlin/NF2 inactivation in tumor biology. Oncogene. 2016;35(5):537-548. 10.1038/onc.2015.125 25893302 PMC4615258

[11] Evans DGR. Neurofibromatosis type 2 (NF2): a clinical and molecular review. Orphanet J Rare Dis. 2009;4:16-16. 10.1186/1750-1172-4-16 19545378 PMC2708144

[12] Maitra S , Kulikauskas RM, Gavilan H, Fehon RG. The tumor suppressors Merlin and expanded function cooperatively to modulate receptor endocytosis and signaling. Curr Biol. 2006;16(7):702-709. 10.1016/j.cub.2006.02.063 16581517

[13] Ramesh V. Merlin and the ERM proteins in Schwann cells, neurons and growth cones. Nat Rev Neurosci. 2004;5(6):462-470. 10.1038/nrn1407 15152196

[14] Weinstock M , McDermott D. Targeting PD-1/PD-L1 in the treatment of metastatic renal cell carcinoma. Ther Adv Urol. 2015;7(6):365-377. 10.1177/1756287215597647 26622321 PMC4647139

[15] Aggen DH , Drake CG, Rini BI. Targeting PD-1 or PD-L1 in metastatic kidney cancer: combination therapy in the first-line setting. Clin Cancer Res. 2020;26(9):2087-2095. 10.1158/1078-0432.CCR-19-3323 31948999

[16] Davis AA , Patel VG. The role of PD-L1 expression as a predictive biomarker: an analysis of all US Food and Drug Administration (FDA) approvals of immune checkpoint inhibitors. J ImmunoTher Cancer. 2019;7(1):278-278. 10.1186/s40425-019-0768-9 31655605 PMC6815032

[17] Thompson RH , Kuntz SM, Leibovich BC, et al. Tumor B7-H1 is associated with poor prognosis in renal cell carcinoma patients with long-term follow-up. Cancer Res. 2006;66(7):3381-3385. 10.1158/0008-5472.CAN-05-4303 16585157

[18] Motzer RJ , Escudier B, McDermott DF, et al. Nivolumab versus everolimus in advanced renal-cell carcinoma. N Engl J Med. 2015;373(19):1803-1813. 10.1056/nejmoa1510665 26406148 PMC5719487

[19] Motzer RJ , Tannir NM, McDermott DF, et al. Nivolumab plus ipilimumab versus sunitinib in advanced renal-cell carcinoma. N Engl J Med. 2018;378(14):1277-1290. 29562145 10.1056/NEJMoa1712126 PMC5972549

[20] Rini BI , Plimack ER, Stus V, et al. Pembrolizumab plus axitinib versus sunitinib for advanced renal-cell carcinoma. N Engl J Med. 2019;380(12):1116-1127. 10.1056/nejmoa1816714 30779529

[21] Motzer RJ , Penkov K, Haanen J, et al. Avelumab plus axitinib versus sunitinib for advanced renal-cell carcinoma. N Engl J Med. 2019;380(12):1103-1115. 10.1056/nejmoa1816047 30779531 PMC6716603

[22] Frampton GM , Fichtenholtz A, Otto GA, et al. Development and validation of a clinical cancer genomic profiling test based on massively parallel DNA sequencing. Nat Biotechnol. 2013;31(11):1023-1031. 10.1038/nbt.2696 24142049 PMC5710001

[23] Chalmers ZR , Connelly CF, Fabrizio D, et al. Analysis of 100,000 human cancer genomes reveals the landscape of tumor mutational burden. Genome Med. 2017;9(1):34-34. 10.1186/s13073-017-0424-2 28420421 PMC5395719

[24] Trabucco SE , Gowen K, Maund SL, et al. A novel next-generation sequencing approach to detecting microsatellite instability and pan-tumor characterization of 1000 microsatellite instability-high cases in 67,000 patient samples. J Mol Diagn. 2019;21(6):1053-1066. 10.1016/j.jmoldx.2019.06.011 31445211 PMC7807551

[25] Venur VA , Santagata S, Galanis E, Brastianos PK. New molecular targets in meningiomas: the present and the future. Curr Opin Neurol. 2018;31(6):740-746. 10.1097/wco.0000000000000615 30379704

[26] Quetel L , Meiller C, Assié JB, et al. Genetic alterations of malignant pleural mesothelioma: association with tumor heterogeneity and overall survival. Mol Oncol. 2020;14(6):1207-1223. 10.1002/1878-0261.12651 32083805 PMC7266286

[27] Pal SK , Choueiri TK, Wang K, et al. Characterization of clinical cases of collecting duct carcinoma of the kidney assessed by comprehensive genomic profiling. Eur Urol. 2016;70(3):516-521. 10.1016/j.eururo.2015.06.019 26149668

[28] Pal SK , Ali SM, Yakirevich E, et al. Characterization of clinical cases of advanced papillary renal cell carcinoma via comprehensive genomic profiling. Eur Urol. 2018;73(1):71-78. 10.1016/j.eururo.2017.05.033 28592388

[29] Yakirevich E , Perrino C, Necchi A, et al. NF2 mutation-driven renal cell carcinomas (RCC): A comprehensive genomic profiling (CGP) study. J Clin Oncol. 2020;38(6_suppl):726-726. 10.1200/jco.2020.38.6_suppl.726

[30] Malouf GG , Ali SM, Wang K, et al. Genomic characterization of renal cell carcinoma with sarcomatoid dedifferentiation pinpoints recurrent genomic alterations. Eur Urol. 2016;70(2):348-357. 10.1016/j.eururo.2016.01.051 26895810

[31] Chen Y-B , Xu J, Skanderup AJ, et al. Molecular analysis of aggressive renal cell carcinoma with unclassified histology reveals distinct subsets. Nat Commun. 2016;7:13131-13131. 10.1038/ncomms13131 27713405 PMC5059781

[32] Mehra R , Vats P, Cieslik M, et al. Biallelic alteration and dysregulation of the hippo pathway in mucinous tubular and spindle cell carcinoma of the kidney. Cancer Discov. 2016;6(11):1258-1266. 10.1158/2159-8290.CD-16-0267 27604489 PMC5096979

[33] Hsieh JJ , Chen D, Wang PI, et al. Genomic biomarkers of a randomized trial comparing first-line everolimus and sunitinib in patients with metastatic renal cell carcinoma. Eur Urol. 2017;71(3):405-414. 10.1016/j.eururo.2016.10.007 27751729 PMC5431298

[34] Fisher R , Horswell S, Rowan A, et al. Development of synchronous VHL syndrome tumors reveals contingencies and constraints to tumor evolution. Genome Biol. 2014;15(8):433-433. 10.1186/s13059-014-0433-z 25159823 PMC4166471

[35] Malouf GG , Flippot R, Dong Y, et al. Molecular characterization of sarcomatoid clear cell renal cell carcinoma unveils new candidate oncogenic drivers. Sci Rep. 2020;10(1):701-701. 10.1038/s41598-020-57534-5 31959902 PMC6971072

[36] Kukuyan AM , Sementino E, Kadariya Y, et al. Inactivation of Bap1 cooperates with losses of Nf2 and Cdkn2a to drive the development of pleural malignant mesothelioma in conditional mouse models. Cancer Res. 2019;79(16):4113-4123. 10.1158/0008-5472.CAN-18-4093 31151962 PMC6697648

[37] Williams EA , Santagata S, Wakimoto H, et al. Distinct genomic subclasses of high-grade/progressive meningiomas: NF2-associated, NF2-exclusive, and NF2-agnostic. Acta Neuropathol Commun. 2020;8(1):171-171. 10.1186/s40478-020-01040-2 33087175 PMC7580027

[38] Ugurluer G , Chang K, Mayeda M, et al. A comprehensive genome-based mutational analysis by next generation sequencing technology in patients with malignant pleural and peritoneal mesothelioma. Int J Rad Oncol Biol Phys. 2015;93(3):S185-S186.

[39] Ammendola S , Simbolo M, Ciaparrone C, et al. Intraventricular meningiomas: clinical-pathological and genetic features of a monocentric series. Curr Oncol. 2022;29(1):178-185. 10.3390/curroncol29010017 35049691 PMC8775267

[40] Badhai J , Pandey GK, Song JY, et al. Combined deletion of Bap1, Nf2, and Cdkn2ab causes rapid onset of malignant mesothelioma in mice. J Exp Med. 2020;217(6):e20191257-e20191257. 32271879 10.1084/jem.20191257 PMC7971132

[41] Shapiro IM , Kolev VN, Vidal CM, et al. Merlin deficiency predicts FAK inhibitor sensitivity: a synthetic lethal relationship. Sci Transl Med. 2014;6(237):237ra268-237ra268. 10.1126/scitranslmed.3008639 PMC4165339 24848258

[42] Pachow D , Andrae N, Kliese N, et al. mTORC1 inhibitors suppress meningioma growth in mouse models. Clin Cancer Res. 2013;19(5):1180-1189. 10.1158/1078-0432.CCR-12-1904 23406776

[43] Bush ML , Burns SS, Oblinger J, et al. Treatment of vestibular schwannoma cells with ErbB inhibitors. Otol Neurotol. 2012;33(2):244-257. 10.1097/MAO.0b013e31823e287f 22222570 PMC3522123

[44] Fuse MA , Dinh CT, Vitte J, et al. Preclinical assessment of MEK1/2 inhibitors for neurofibromatosis type 2-associated schwannomas reveals differences in efficacy and drug resistance development. Neuro Oncol. 2019;21(4):486-497. 10.1093/neuonc/noz002 30615146 PMC6422635

[45] Lang GT , Jiang YZ, Shi JX, et al. Characterization of the genomic landscape and actionable mutations in Chinese breast cancers by clinical sequencing. Nat Commun. 2020;11(1):5679-5679. 10.1038/s41467-020-19342-3 33173047 PMC7656255

[46] Sourbier C , Liao PJ, Ricketts CJ, et al. Targeting loss of the Hippo signaling pathway in NF2-deficient papillary kidney cancers. Oncotarget. 2018;9(12):10723-10733. 10.18632/oncotarget.24112 29535838 PMC5828210

[47] McDermott DF , Lee JL, Bjarnason GA, et al. Open-label, single-arm phase II study of pembrolizumab monotherapy as first-line therapy in patients with advanced clear cell renal cell carcinoma. J Clin Oncol. 2021;39(9):1020-1028. 10.1200/JCO.20.02363 33529051 PMC8078336

[48] Powles T , Plimack ER, Soulières D, et al. Pembrolizumab plus axitinib versus sunitinib monotherapy as first-line treatment of advanced renal cell carcinoma (KEYNOTE-426): extended follow-up from a randomised, open-label, phase 3 trial. Lancet Oncol. 2020;21(12):1563-1573. 10.1016/S1470-2045(20)30436-8 33284113

[49] Choueiri TK , Powles T, Burotto M, et al; CheckMate 9ER Investigators. Nivolumab plus cabozantinib versus sunitinib for advanced renal-cell carcinoma. N Engl J Med. 2021;384(9):829-841. 10.1056/NEJMoa2026982 33657295 PMC8436591

[50] Kinoshita Y , Hamasaki M, Yoshimura M, et al. Hemizygous loss of NF2 detected by fluorescence in situ hybridization is useful for the diagnosis of malignant pleural mesothelioma. Mod Pathol. 2020;33(2):235-244. 10.1038/s41379-019-0309-6 31231129

