# Supplementary material for: Comprehensive Genomic Profiling of NF2-Mutated Kidney Tumors Reveals Potential Targets for Therapy
Source: Oncologist. 2023 Mar 14;28(7):e508–19. doi: 10.1093/oncolo/oyad040 (PMC10322144; doi:10.1093/oncolo/oyad040)
Supplement: oyad040_suppl_Supplementary_Tables [file oyad040_suppl_supplementary_tables.docx]

**Supplemental Table 1.** Demographic patient data for total cohort of kidney tumors.

| **Kidney tumor** | ***n*** | **Female (%)** | **Male (%)** | **Age, yrs**  **(median)** | **Age, yrs**  **(range)** |
| --- | --- | --- | --- | --- | --- |
| Unclassified renal cell carcinoma | 197 | 64 (32.99) | 133 (67.51%) | 62 | (12 - >89) |
| Chromophobe renal cell carcinoma | 108 | 38 (35.19) | 70 (64.81%) | 58 | (15 - 85) |
| Clear cell renal cell carcinoma | 1875 | 548 (29.23) | 1327 (70.77%) | 60 | (17 - >89) |
| Collecting duct carcinoma | 61 | 18 (29.51) | 43 (70.49%) | 55 | (26 - 87) |
| Medullary carcinoma | 49 | 22 (44.90) | 27 (55.10%) | 27 | (8 - 65) |
| Papillary renal cell carcinoma | 405 | 88 (21.73) | 317 (78.27%) | 63 | (19 - >89) |
| Sarcomatoid carcinoma | 171 | 56 (32.75) | 115 (67.25%) | 60 | (26 - >89) |
| Urothelial carcinoma | 906 | 365 (40.29) | 541 (59.71%) | 70 | (30 - >89) |
| Wilms tumor | 147 | 83 (56.46) | 64 (43.54%) | 6 | (0 - 55) |
| **Total** | **3919** | **1282 (32.71)** | **2637 (67.29%)** | **62** | **(0 - >89)** |

**Supplemental Table 2.** Validation of the top 30 genes from the Foundation Medicine cohort in in the TCGA cohort.

| Co-mutation | *NF2*wt type (n =1451) | *NF2*mut (n = 35) | Log rank | p value | Enriched |
| --- | --- | --- | --- | --- | --- |
| *VHL* | 400 (27.57%) | 4 (11.43%) | -1.12 | **0.0393** | wt |
| *PBRM1* | 257 (17.71%) | 3 (8.57%) | -0.92 | 0.157 |  |
| *CDKN2A* | 34 (2.34%) | 4 (11.43%) | 1.30 | 0.090 | - |
| *TP53* | 88 (6.06%) | 2 (5.71%) | 0.10 | 0.100 | - |
| *TERT* | 21 (1.45%) | 2 (5.71%) | 1.77 | 0.127 | - |
| *CDKN2B* | 28 (1.93%) | 4(11.43%) | 1.58 | 0.0536 | - |
| *SETD2* | 173 (11.92%) | 12 (34.29%) | 1.27 | **3.284e-3** | mut |
| *BAP1* | 142 (9.79%) | 9 (25.71%) | 1.01 | **0.0344** | mut |
| *ARIDA1* | - (0.00%) | - (0.00%) |  | - | - |
| *PIK3CA* | 37 (2.55%) | 1 (2.86%) | 0.22 | 0.587 | **-** |
| *PTEN* | 57 (3.93%) | 0 (0.00%) | **<**-10 | 0.357 | **-** |
| *KDM5C* | 63 (4.34%) | 0 (0.00%) | <-10 | 0.305 | - |
| *MTAP* | 19 (1.31%) | 3 (8.57%) | 2.61 | **0.016** | mut |
| *FGFR3* | 23 (1.59%) | 1 (2.86%) | 0.95 | 0.417 | **-** |
| *KDM6A* | 18 (1.24%) | 2 (5.71%) | 1.60 | 0.155 | - |
| *TSC1* | 24 (1.65%) | 0 (0.00%) | <-10 | 0.683 | - |
| *KMT2D* | 51 (3.51%) | 0 (0.00%) | <-10 | 0.451 | - |
| *MDM2* | 19 (1.31%) | 0 (0.00%) | <-10 | 0.693 | **-** |
| *CREBBP* | 21 (1.45%) | 2 (5.71%) | 1.80 | 0.122 | - |
| *ATM* | 40 (2.76%) | 0 (0.00%) | <-10 | 0.546 | - |
| *CCND1* | 3 (0.21%) | 0 (0.00%) | <-10 | 0.878 | - |
| *TET2* | 24 (1.65%) | 1 (2.86%) | 0.75 | 0.460 | - |
| *FGF19* | 5 (0.34%) | 0 (0.00%) | <-10 | 0.796 | - |
| *RB1* | 10 (0.69%) | 1 (2.86%) | 2.09 | 0.227 | - |
| *KRAS* | 9 (0.62%) | 0 (0.00%) | <-10 | 0.834 | - |
| *DNMT3A* | 29 (2.00%) | 2 (5.71%) | 2.03 | 0.092 | - |
| *SMARCB1* | 32 (2.21%) | 3 (8.57%) | 1.82 | 0.058 | - |
| *MTOR* | 67 (4.62%) | 1 (2.86%) | -0.54 | 0.570 | - |
| *FGF3* | 5 (0.34%) | 0 (0.00%) | <-10 | 0.796 | - |
| *MYC* | 9 (0.62%) | 0 (0.00%) | <-10 | 0.923 | - |
