# Supplementary material for: Comprehensive Genomic Profiling of NF2-Mutated Kidney Tumors Reveals Potential Targets for Therapy
Source: Oncologist. 2023 Mar 14;28(7):e508–19. doi: 10.1093/oncolo/oyad040 (PMC10322144; doi:10.1093/oncolo/oyad040)
Supplement: oyad040_suppl_Supplementary_Figure [file oyad040_suppl_supplementary_figure.docx]

**Supplemental Figure 1.** Top 50 co-mutated genes across all kidney tumor subtypes.

(a) ccRCC, (b) pRCC, (c) sRCC, (d) cdRCC, (e) mRCC, (f) uRCC, (g) UC, and (h) Wilms tumors.
